# Relationship between blood glucose and carotid intima media thickness: A meta-analysis

**DOI:** 10.1186/1475-2840-9-37

**Published:** 2010-08-13

**Authors:** Thomas R Einarson, Jonathan Hunchuck, Michiel Hemels

**Affiliations:** 1 Leslie Dan Faculty of Pharmacy, University of Toronto, Toronto, Canada; 2 HTA Intelligence unit, Global Health Economics & Outcomes Research, NovoNordisk A/S, Bagsværd, Denmark

## Abstract

**Background:**

Increased coronary intima media thickness (CIMT) has been associated with adverse cardiovascular outcomes, as have increased glucose levels. The link has not been established between glucose and CIMT; therefore, we sought to assess the relationship between glucose and CIMT.

**Methods:**

Medline, EMBASE, Scopus, and Cochrane databases were searched from inception through 2009 for original research reporting both postprandial glucose levels and CIMT measurements. Glucose was classified as normal, impaired, or diabetic. Outputs included inverse variance weighted effect size and also average correlation (using the Wang and Bushman approach). Data were combined using a random effects meta-analytic model. Heterogeneity as assessed using χ^2 ^and I^2^; bias was examined using Egger plots and Begg-Mazumdar tau. Polynomial functions (i.e., linear, quadratic, cubic, quartic) were fit to the data and the Akaike Information Criteria were used to select the optimal model.

**Results:**

We identified 172 papers; 161 were rejected (19 inappropriate design, 8 had selected patients, 101 inappropriate outcomes) leaving 11 accepted. We used data from 15,592 patients (8250 normals, 3013 impaired glucose, 4329 diabetics). There was no evidence of heterogeneity or publication bias. The overall correlation was 0.082 (CI_95%_:0.066-0.098); the overall effect size was 0.294 (0.245-0.343) between diabetics and normals and 0.137 (0.072-0.202) between normals and those with impaired glucose. The equation of best fit was linear (CIMT = 0.828 + 0.009*glucose).

**Conclusions:**

There is a small but significant relationship between postprandial glucose levels and CIMT, which have both been associated with adverse cardiovascular outcomes.

## Background

The National Institutes of Health defines atherosclerosis as "an arteriosclerosis characterized by atheromatous (i.e., fatty) deposits in and fibrosis of the inner layer of the arteries"[1]. The major locus for the formation of atherosclerotic plaques is in the carotid arteries. The problem starts at the point where the common carotid artery bifurcates into the internal and external branches. Progression occurs mainly along the proximal part of the common carotid artery and into the proximal section of the internal carotid artery.

In middle aged men, the intima and media of the common carotid artery together measure from 0.7-1.2 mm[2]. The American Heart Association also noted that the arterial intima is not of uniform thickness, with normal arteries having an intima: media ratio of 0.1 to 1.0, or even more. Increases in carotid intima media thickness (CIMT) are associated with a number of factors, including age, sex, hypertension, smoking, lipid profile, and body mass index[3]. Lorenz et al.[4] have referred to CIMT as "an intermediate phenotype for early atherosclerosis". This marker for disease has an advantage in that it can be measured rather easily with ultrasonography, which is also non-invasive[5]. Therefore, it may be used to screen large groups of patients.

Ceriello[6] has linked hyperglycemia to cardiovascular disease through a series of relationships. He proposed that hyperglycemia creates oxidative and nitrosative stress, which act on the arterial wall to initiate the thickening process. Brohall and associates[7] reported that impaired glucose tolerance was not associated with atherosclerosis, but it was associated with intima medial thickness. Epidemiologic support for an overall relationship was produced in 1999 by Coutinho and coworkers[8] who performed a meta-analysis of studies to date. They found a positive relationship between postprandial glucose levels in non-diabetics and the later development of cardiovascular events (CVEs), including myocardial infarction, stroke, and death. In 2007, Lorenz and coworkers[4] published a meta-analysis that investigated the relationship between CIMT and those same CVEs. They concluded that CIMT strongly predicted future vascular events, with a relative risk per CIMT difference that was somewhat greater for stroke than for myocardial infarction.

A search of the literature could find no studies that have definitively quantified the link between glucose levels and CIMT. If the theory proposed by Ceriello is correct, then there should be supporting evidence from epidemiologic studies. Therefore, the purpose of the present research was to quantify the relationship between blood glucose levels and CIMT. There were two specific aspects of interest, which were inference and modeling. The inferential analysis focused on estimating the correlation between CIMT and glucose and to test whether this relationship was statistically significant. The modeling analysis focused on fitting a series of regression models to the dataset and choosing the optimal model to represent the data.

## Methods

We sought to retrieve all research studies of persons who had both CIMT measurements and blood glucose levels. Patients could have any glucose status, but the studied sample could not consist exclusively of diabetics; there must have been at least one group of non-diabetics. Papers could be published in any language at any time. The following databases were searched from the date they started until the end of 2009: Medline, EMBASE, Scopus, and Cochrane. Search terms included "glucose" or "blood sugar" and "CIMT" or "coronary artery media" or "stenosis" or "medial thickness", and combinations thereof. Two reviewers performed the search and a third adjudicated discrepancies and independently verified all steps of the process.

Data extracted included the means and standard deviations for CIMT and 2-hour post prandial glucose levels. For data analysis, we classified glucose levels by groups corresponding to the categories of diabetes as defined by the American Diabetes Association[9], which were normal, impaired, and diabetic. If glucose levels by group were unavailable, the boundary values (7.8, 9.45, and 11.1 mmol/L) were imputed. If the article reported more than three subgroups related to glucose levels, the weighted averages of the estimates (using means and standard deviations) from the relevant subgroups were computed.

Using these means and standard deviations, and assuming a normal distribution, five hundred datasets per study were simulated and the average correlation coefficient and related confidence interval were computed per study[10]. Applying the method from Wang and Bushman[11], the overall estimate for the correlation (i.e., Pearson's r) and its confidence interval were computed. The macros 'wavgmeta' and 'covtefst' were used to compute the weighted average correlation estimates[11,12]. A forest plot was used to depict and summarize the individual and overall correlations.

In order to model the overall relationship between CIMT and glucose over the range of reported glucose levels, a series of polynomial functions (i.e. linear, quadratic, cubic, quartic) were fit to the data[13,14]. The Akaike Information Criterion (AIC) were used to select the optimal model. It is calculated as AIC = 2k - 2 ln(L), with k being the number of parameters and L being the maximized value of the likelihood function for the estimated model. This index is used to select between competing models. The original application was for time-series models, but it may also be applied to regression models. The AIC considers both the goodness of fit and number of parameters in the model, with lower values of the index (which are associated with the fewest parameters) being preferred.

In addition to the correlation analysis, effect sizes were calculated to quantify the relationship between the diabetic group and the normal group as well as between the impaired group and the normal group. Forest plots were used to summarize the computed effect sizes and related confidence intervals. To test for heterogeneity between studies, the Cochran Q test[15] and the I^2 ^tests[16,17] were performed. In order to test for bias, the Egger[18] and Begg-Mazumdar[19] tests were performed. The software packages used in this analysis were SAS (SAS Institute Inc., Cary, NC) and StatsDirect (StatsDirect Ltd., Cheshire, UK).

## Results

The initial screening identified 172 potential papers, of which 161 were rejected (19 had inappropriate study designs, 8 dealt with specific selected disease states, and 134 had inappropriate outcomes (101 did not report glucose levels in ≥3 quantiles, 24 did not indicate the numbers of outcomes within each quantile, and 9 did not report CIMT values). That left 11 acceptable studies for the analysis, which are summarized in Table 1[20-30].

**Table 1:** Characteristics of the accepted studies and correlation of post prandial glucose level with CIMT.

		Number of persons studied					Correlation coefficient
				
		Glucose category				Glucose	Pearson's r
					
Author	Year	normal	impaired	diabetic	Total	Groups*	(95% CI)
Faeh[1]	2007	776	150	184	1,110	3	0.075 (0.017-0.133)
Henry[2]	2004	278	168	301	747	3	0.094 (0.022-0.164)
Hunt[3]	2003	1,127	66	303	1,496	3	0.124 (0.074-0.174)
Ishizaka[4]	2003	738	334	166	1,238	3	0.056 (0-0.164)
Mohan[5]	2006	1,600	330	1,500	3,430	4	0.086 (0.053-0.119)
O'Leary[6]	1992	2,576	1,427	1,161	5,164	3	0.069 (0.042-0.096)
Niskanen[7]	1996	98	21	84	203	3	0.104 (-0.034-0.238)
Rajala[8]	2002	57	97	54	208	3	0.085 (-0.051-0.218)
Temelkova[9]	2000	265	82	88	435	4	0.180 (0.088-0.270)
Tuomilehto[10]	1998	100	25	44	169	3	-0.020 (-0.169-0.131)
Wagenknecht[11]	1998	635	313	444	1,392	4	0.077 (0.024-0.129)
							
Overall		8,250	3,013	4,329	15,592		0.082 (0.066-0.098)

*Indicates the number of glucose categories reported. In the Temelkova study, four groups were reported; however, only three were used for the analysis

Correlation coefficients are presented in Table 1. Their values ranged from -0.02 to 0.180, with an average of 0.082 (CI95%: 0.061-0.094), which is small but indicates a significant relationship that is beyond chance. The tests for heterogeneity were non-significant (Cochran's Q = 10.44, P = 0.40; I^2 ^= 4.2%). Egger's test found no evidence of bias (intercept = 0.55, P = 0.59), as did the Begg-Mazumdar statistic (τ = 0.052; P = 0.52). Therefore, results were considered combinable.

**Figure 1:**
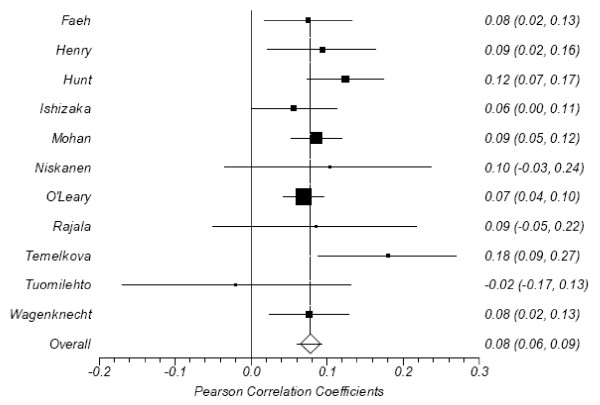
**Forest plot of correlations between CIMT and postprandial glucose**.

The results of the correlation analysis (per study and overall) are listed in Table 1 and depicted in Figure 1. The correlations coefficients from 8 (73%) of the 11 articles were positive and statistically significant, as was the overall correlation (r = 0.082; 95% confidence limits 0.066, 0.098). Cohen[31] would define that value as small, but positive.

**Figure 2:**
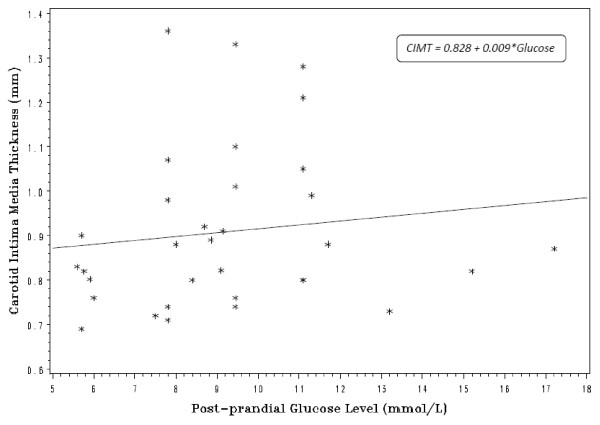
**Equation of best fit for the relationship between CIMT and glucose**.

A series of polynomial regression models were then fit to the glucose and carotid intima media thickness estimates. Using the AIC criteria, the linear model (Y = α + β*X; Y = CIMT, X = Glucose, α = 0.828, β = 0.009) was chosen as having the best fit, since it had the lowest value of -6.5. The relationship between carotid intima media thickness is plotted in Figure 2 with the regression function overlaid.

**Figure 3:**
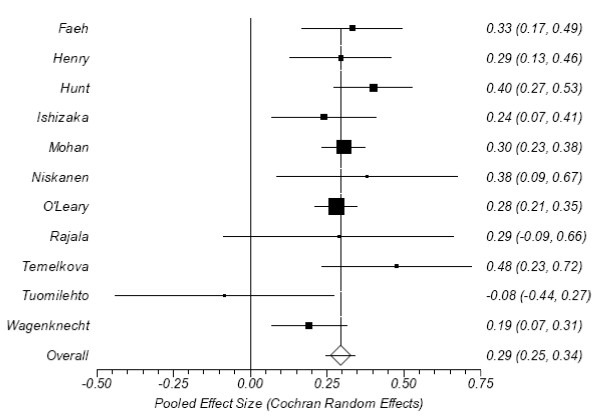
**Forest plot of effect sizes between CIMT and post prandial glucose levels in diabetics versus normals**.

**Figure 4:**
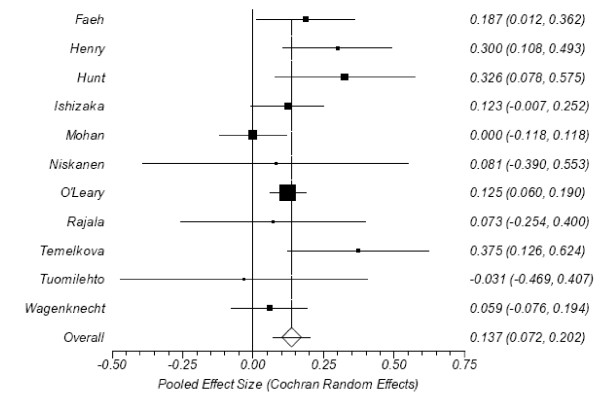
**Forest plot of effect sizes between CIMT and post prandial glucose levels in persons with impaired glucose tolerance versus normals**.

The calculated effect sizes from the accepted studies are presented in Table 2; forest plots appear in Figures 3 and 4. All tests for heterogeneity were non-significant (I^2 ^= 24% and Cochran's Q = 13.07, P = 0.22 for normals; I^2 ^= 37% and Q = 15.85, P = 0.10 for impaired glucose. Egger's test found no evidence of publication bias (intercepts = -0.08, P = 0.91 and 0.60, P = 0.44, respectively), nor did the Begg-Mazumdar test (τ = -0.05, P = 0.76 and τ = 0.13, P = 0.65, respectively). The overall effect size between diabetics and normals was 0.294 (CI95%: 0.245-0.343), which Cohen[31] would define as small. The effect size between those with impaired glucose and normals was 0.137 (CI95%: 0.072-0.202), which is also small, but also significant.

**Table 2:** Effect sizes (95% confidence limits) for the difference in CIMT between categories of post prandial glucose levels.

	Glucose categories being compared	
	
	Diabetic-Normal	Impaired-Normal
Faeh[1]	0.331 (0.170, 0.493)	0.187 (0.012, 0.362)
Henry[2]	0.293 (0.129, 0.457)	0.300 (0.107, 0.492)
Hunt[3]	0.401 (0.273-0.528)	0.326 (0.078-0.575)
Ishizaka[4]	0.239 (0.071, 0.408)	0.122 (-0.006, 0.252)
Mohan[5]	0.304 (0.233, 0.375)	0 (-0.118, 0.118)
O'Leary[6]	0.279 (0.210, 0.349)	0.125 (0.060, 0.189)
Niskanen[7]	0.379 (0.085, 0.673)	0.081 (-0.390, 0.552)
Rajala[8]	0.288 (-0.085, 0.662)	0.072 (-0.254, 0.399)
Temelkova[9]	0.477 (0.233, 0.721)	0.374 (0.125, 0.623)
Tuomilehto[10]	-0.082 (-0.437, 0.271)	-0.030 (-0.469, 0.407)
Wagenknecht[11]	0.192 (0.070, 0.313)	0.058 (-0.076, 0.194)
		
Overall	0.294 (0.245, 0.343)	0.137 (0.072, 0.202)

## Discussion

In a very thorough review of the evidence based literature, Helfand et al. 2009[32] found that there were as yet no accepted categories that could be effectively used for cardiac risk assessment. They also noted that epidemiologic studies used a variety of measurements, but that there was no established consensus for which was the best for assessing cardiovascular risk.

Unlike other studies that have examined glucose and its relationship to subsequent cardiovascular outcomes, the data pool in our study was much more restricted due to the nature of the screening process. That is, CIMT imaging is only done when there is a reason to do so; seldom were there random screenings of the general population. Most likely reasons include the expense involved and time commitment. As well, the measurement of CIMT does not have a long history. It has only been done since Poli and coworkers initially described the technique in 1988[5].

This analysis indicates there is significant relationship between intima medial thickness and glucose levels. The overall correlation (r = 0.082) was positive and the confidence interval did not include zero. Similarly, of the individual studies, 8 of the 11 (73%) were positive and statistically significant (i.e., the confidence intervals do not include zero). The absolute value of the correlation and effects sizes is not large, but that is to be expected from the type of data that we were able to utilize in the analysis. The glucose values were reported in categories; if actual means had been available, the estimates would have been more precise.

The regression analysis identified a modest positive relationship between CIMT and post-prandial glucose levels. The regression line plot indicates the positive linear relationship between these two factors, and the linear regression function is preferred to the higher order functions. Again, it has similar limitations due to the measurement of the outcome data.

The conclusions of the effect size analysis are consistent with the conclusions of the correlation analysis. There was a positive and statistically significant relationship between CIMT and post-prandial glucose levels. Tests for heterogeneity and bias did not indicate the presence of these factors.

These findings confirm those of Ceriello[6] and establish that there is a statistically significant link between post prandial glucose levels and CIMT. Previous reviews have established that increased CIMT does lead to adverse cardiovascular outcomes. In 2002, Cheng's group published a review of all of the available studies finding a positive relationship in more than 2000 patients[33]. Furthermore, when the CIMT was reduced, cardiovascular events decreased. A larger review by Brohall and colleagues[34] in 2006 examined 24,111 patients in 23 studies of type-2 diabetics and persons with abnormal glucose tolerance. They found a 40% increase in strokes among diabetics and a smaller increase in glucose intolerant persons. The strongest quantitative evidence was provided in 2007 by Lorenz and coworkers[4], who published a meta-analysis of data from 37,197 patients in 8 studies that were described in 12 papers. For every 0.1 mm increase in CIMT, there was a 10% increase in the risk of myocardial infarction and a 13-18% increase in the risk for stroke.

More recently, a clinical study in type-2 diabetics by Djaberi et al.[35] reported a significant relationship between CIMT and abnormal cardiac perfusion. These results could help explain the sequence of events. Other recent publications provide additional information, including papers by Ito and coworkers[36], Escobedo and colleagues[37], and Poppe and associates[38]. These articles confirm that vascular imaging can be effectively used to detect subclinical disease in type 2 diabetics and potentially predict cardiovascular risk. Escobedo's group[37] found that abnormalities in CIMT were widespread in Latin America, suggesting that screening might be an option. Ito's study[36] established the relationship between increased CIMT and glomerular filtration rate, a marker for kidney disease, as well as diabetic nephropathy. Finally, the Poppe group[37] determined that CIMT and carotid plaque increased in concert with the number of factors associated with metabolic syndrome. Such findings are in agreement with what we have reported in the present research.

Among the limitations of this study is the small number of studies available for analysis. Ideally, larger numbers are preferable, but these are what could be found in the literature. Nonetheless, we did analyze data from more than 15,000 patients, which does afford some degree of confidence in the findings. As well, we examined only post prandial glucose levels. It may well be that fasting levels would produce more robust results. Another option would be to examine the relationship between glycosylated haemoglobin and CIMT. In addition, we were not able to adjust for potential confounders, as they were not uniformly reported across all studies, rendering meta-regression non-feasible.

## Conclusions

We have demonstrated that a significant relationship exists between post prandial glucose levels and CIMT. These events, in turn, have been associated with adverse cardiovascular events such as stroke and myocardial infarction. Since the measurement of CIMT is non-invasive, it may prove to be a useful tool both in diagnosing potential problems, but also in monitoring treatments and their outcomes.

## Abbreviations

AIC: Akaike Information Criterion; CI: confidence interval; CIMT: carotid intima media thickness; CVE: cardiovascular event; ln: natural logarithm

## Competing interests

This manuscript does not deal with any specific product, rather, it investigates the relationship between glucose levels and CIMT. Therefore, competing interests could arise with companies who sell products aimed at detecting or measuring glucose or related compounds (e.g., HbA1c) in the body, reducing blood glucose (e.g., insulin or oral hypoglycemic agents), or measuring CIMT.

TRE has directly or indirectly consulted with a variety of firms who manufacture or are developing ant diabetic drugs or testing devices (i.e., NovoNordisk, Generex, Bristol-Myers-Squibb, Bayer). He received consulting funds for the undertaking of this project.

JH has no conflicts.

MH is an employee of NovoNordisk who provided the funding for this project.

## Authors' contributions

TRE was the overall coordinator and primary writer of the manuscript. He performed literature searches, data extraction and validation, and interpretation of results.

JH performed literature searches, data extraction and validation, and data analysis. He also participated in the writing of the paper.

MH conceived of the study, and participated in its design and coordination and helped to draft the manuscript. All authors read and approved the final manuscript.
